# Effectiveness of nasal highflow in hypercapnic COPD patients is flow and leakage dependent

**DOI:** 10.1186/s12890-018-0576-x

**Published:** 2018-01-24

**Authors:** Jens Bräunlich, Friederike Mauersberger, Hubert Wirtz

**Affiliations:** Department of Respiratory Medicine, University of Leipzig, Liebigstrasse 20, 04103 Leipzig, Germany

**Keywords:** COPD, Hypercapnia, Nasal high flow, Type II respiratory failure, Wash-out

## Abstract

**Background:**

Nasal Highflow (NHF) delivers a humidified and heated airflow via nasal prongs. Current data provide evidence for efficacy of NHF in patients with hypoxemic respiratory failure. Preliminary data suggest that NHF may decrease hypercapnia in hypercapnic respiratory failure. The aim of this study was to evaluate the mechanism of NHF mediated PCO_2_ reduction in patients with chronic obstructive pulmonary disease (COPD).

**Methods:**

In 36 hypercapnic COPD patients (PCO_2_ > 45 mmHg), hypercapnia was evaluated by capillary gas sampling 1 h after NHF breathing under four conditions A to D with different flow rates and different degrees of leakage (A = 20 L/min, low leakage, two prongs, both inside; B = 40 L/min, low leakage, two prongs, both inside; C = 40 L/min, high leakage, two prongs, one outside and open; D = 40 L/min, high leakage, two prongs, one outside and closed). Under identical conditions, mean airway pressure was measured in the hypopharynx of 10 COPD patients.

**Results:**

Hypercapnia significantly decreased in all patients. In patients with capillary PCO_2_ > 55 mmHg (*n* = 26), PCO_2_ additionally decreased significantly by increased leakage and/or flow rate in comparison to lower leakage/ flow rate conditions (A = 94.2 ± 8.2%; B = 93.5 ± 4.4%; C = 90.5 ± 7.2%; D = 86.8 ± 3.8%). The highest mean airway pressure was observed in patients breathing under condition B (2.3 ± 1.6 mbar; *p* < 0.05).

**Conclusions:**

This study demonstrates effective PCO_2_ reduction with NHF therapy in stable hypercapnic COPD patients. This effect does not correlate with an increase in mean airway pressure but with increased leakage and airflow, indicating airway wash out and reduction of functional dead space as important mechanisms of NHF therapy. These results may be useful when considering NHF treatment in hypercapnic COPD patients.

**Trial registration:**

Clinical Trials: NCT02504814; First posted July 22, 2015.

## Background

Ventilatory support systems have become an important therapeutic mean in patients with chronic respiratory disease. These devices create a gradient between atmospheric pressure and airway pressure, thus establishing a driving pressure during inspiration as well as a variable amount of positive end expiratory airway pressure. Respiratory frequency and a minimum minute volume may be specified. This kind of ventilator support improves alveolar ventilation and consecutively reduces PCO_2_ [1]. The aim of non-invasive ventilation support is an improvement of respiratory muscle function by partially relieving respiratory muscle load and, subsequently, the normalization of setpoint values of the sensors of the respiratory system.

A novel development in non-invasive ventilation support is the application of a constant high nasal airflow with 20 to 60 L/min. This technique is known as nasal high flow (NHF) therapy. Patients tolerate flow rates in this order of magnitude when gas is warmed and humidified close to body standard temperature and humidity before entering the nose. In addition, the gas mixture may contain any fraction of oxygen set from 0.21 to 1.0.

NHF therapy has become frequently used in the treatment of neonatal patients as an alternative to nasal continuous positive airway pressure (nCPAP) therapy [2]. However, there are increasing data that demonstrate the benefits of NHF therapy also in adult patients. Maggiore et al. [3] were able to show superiority in oxygen delivery and reintubation rate of NHF therapy over Venturi mask oxygen therapy following extubation of patients with acute respiratory failure. In their study, Frat et al. [4] demonstrated that in patients with primary respiratory failure, NHF therapy is equally effective and partially superior to oxygen supply and non-invasive ventilation (NIV) in terms of reduced intubation rates. In the study of Stéphan et al. [5], NHF therapy was shown not to be inferior to NIV in patients who suffered from respiratory failure after cardiothoracic surgery. Furthermore, Hernandez and colleagues documented benefits in patients with low and high risk of postextubation respiratory failure [6, 7]. The results of these studies emphasize the benefits and the safety of NHF therapy in the treatment of acute hypoxemic respiratory failure. However, when compared to conventional NIV, much less experience is available from clinical studies on NHF therapy. In fact, the standard indications of NIV, acute and chronic hypercapnic respiratory failure, have not been investigated yet.

Few studies have investigated the effect of NHF therapy on e.g. hypercapnia in patients with chronic obstructive pulmonary disease (COPD), but the number of patients recruited in these studies is limited [8–13]. In addition, the mechanisms that may lead to a decrease in hypercapnia have not been elucidated so far. Some authors suggest the increase in airway pressure to be responsible for the effects of NHF therapy [14, 15]. However, the increase in airway pressure seems to be rather small. Another possible explanation focuses on the wash out effect within the upper airways [16, 17]. Frizzola et al. [18] investigated the mechanism of NHF therapy in a piglet model of lung injury. In this model, the reduction of PCO_2_ during NHF, as opposed to the situation in NIV, did not correlate with tracheal pressure but was more efficient, when leakage of gas from the nose was increased. The authors therefore suggested that wash out of nasopharyngeal dead space by nasally applied high flow improves CO_2_ removal in the piglet model. The high flow generated by NHF can pass functional dead space more easily and may therefore economize the work of breathing [19–21]. The aim of this study was therefore to investigate whether the effects of NHF therapy in hypercapnic COPD patients depend on flow rates, airway pressure, and leakage.

## Methods

### Study design/ outcome parameters

This study had a prospective, unblinded, and single-center design. Outcome parameters were changes in capillary PCO_2_, PO_2_, and mean airway pressure during various flow rates and leakage conditions.

### Patients

For this study, 46 patients were recruited in the department of respiratory medicine of the University hospital Leipzig in 2015. The study was approved (414–14-06102014) and registered (ClinicalTrails NCT02504814) by the ethics committee of the Medical Faculty, University of Leipzig. All patients gave written informed consent to participate in the study. Criteria for inclusion in the study were a diagnosis of COPD (GOLD), PCO_2_ > 45 mmHg, chronic ventilatory insufficiency with hypercapnia without additional metabolic alterations, > 18 years of age, and a stable stage of disease. Patients with acute ventilatory insufficiency (pH < 7.35), instable disease (e.g. exacerbation, systemic inflammation, pneumonia), incomplete or untapped medical therapy, other lung diseases, inadequate compliance to any kind of treatment, as well as fertile women and patients participating in other trails were excluded.

### NHF ventilator

In this study, the TNI20s oxy device (TNI medical AG, Wuerzburg, Germany) was used. The system was equipped with an additional humidifier/heater (Hydrate OMNI™, Hydrate Inc., Midlothian, USA) to ensure the intended higher flow rates because the original humidifier was not able to sufficiently humidify when flow rates were higher than 24 L/min. Flow rates were additionally verified with an electronic flowmeter (TSI 4000 Series®, TSI Inc., Shoreview, USA). With these modifications, the NHF system provided stable flow rates of up to 50 L/min and allowed the admixture of oxygen.

### Measuring changes in hypercapnia

Patients were randomly assigned to undergo sequences of treatment conditions. Randomization was computer-generated with a sequence of numbers, and each number was associated with a character (1 = A, 2 = B, 3 = C, 4 = D). Some patients were treated by all conditions. Changes in hypercapnia were measured in 36 COPD patients who had undergone NHF therapy for 1 h. Capillary blood gas samples were taken prior to NHF therapy, 30 min after the beginning, and immediately after completion of one condition at 60 min. The starting PCO_2_ prior to NHF therapy was considered as baseline PCO_2_. If more than one of the four possible NHF conditions were tested in one patient at 1 day, a treatment-free interval of at least 2 h between the two conditions was assured. Note that if patients were given continuous oxygen insufflations within the scope of their regular therapy, oxygen supply was neither altered nor interrupted during the duration of NHF treatment.

**Fig. 1:**
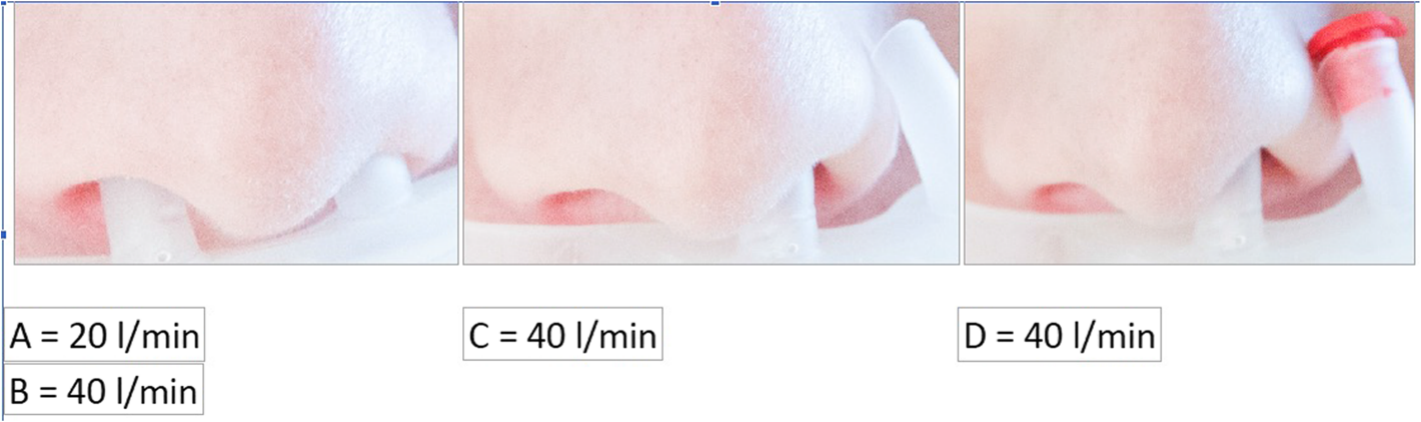
Experimental conditions. A = 20 L/min, low leakage, two prongs, both inside; B = 40 L/min, low leakage, two prongs, both inside; C = 40 L/min, high leakage, two prongs, one outside and open; D = 40 L/min; high leakage, two prongs, one outside and closed

The leakage and flow combinations tested (conditions A – D) were combinations of two different flow rates (20 L/min and 40 L/min) and two degrees of leakage (NHF applied through one or two nostrils; Fig. 1). Note that in condition C, the effective flow rate was 20 L/min with the device set at 40 L/min but with only one of two equal prongs inserted to one nostril.

### Blood gas analysis

Blood samples for capillary blood gas analyses were obtained from a hyperemic earlobe after 10 min of treatment with a vasodilatation ointment (Finalgon®, Boehringer Ingelheim Pharma GmbH & Co. KG, Ingelheim am Rhein, Germany). All measurements were performed with constant oxygen supplementation as indicated.

### Measuring mean airway pressure in COPD patients

Mean airway pressure was measured in 10 patients with COPD. First, a water-filled thin flexible tube (1 mm in diameter, Original Perfusor®-cable type standard, B. Braun, Melsungen, Germany) was connected to a pressure sensor (GMH3111, Greisinger electronic GmbH, Regenstauf, Germany). The free end was inserted in the nasopharynx under visual control. The electronic pressure sensor was hooked to a computer. A total of 10 breaths were recorded during spontaneous breathing (baseline) and another 10 breaths were recorded during each of the experimental conditions.

### Statistics

The sample size of 36 had 85% power, with a type I error rate of 5%, to detect a difference in capillary pCO_2_ of 5%. Data were analyzed using single factor ANOVA. If F > F crit, we rejected the null hypothesis. Additionally, we used Student’s t-test (Sigma Plot, Systat Software GmbH, Ekrath, Germany) to compare each pair of means (in case of rejecting null hypothesis). A *p*-value <0.05 was considered as significant.

## Results

Demographic data are presented in Table 1. NHF significantly reduced PCO_2_ in comparison to baseline in all patients (A: 95.3 ± 8.0%, *n* = 22; B: 95.6 ± 5.8%, *n* = 19; C: 94.5 ± 9.9%, *n* = 20; D: 95.1 ± 8.6%, n = 19). No significant differences were observed between the four experimental conditions with regard to PCO_2_ after treatment.

**Table 1:** Demographic and clinical data of all patients

Characteristics	Study participants (*n* = 36)
Age (years; mean ± SD)	60.5 ± 14.3
Gender male (n): female (n)	19: 17
Oxygen insufflation (L/min; mean ± SD)	1.9 ± 1.4
FEV1 (% predicted; mean ± SD)	29.9 ± 13.3
FEV1%FVC (predicted; mean ± SD)	54.1 ± 17.5
Rtot (% predicted; mean ± SD)	346.9 ± 153.5
TLC (% predicted; mean ± SD)	111.6 ± 21.3
RV (% predicted; mean ± SD)	210.3 ± 78.3
RV%TLC (% predicted; mean ± SD)	183.4 ± 40.2
pH (mean)	7.4029 ± 0,039
PO_2_ (mmHg; mean ± SD)	66.3 ± 18.4
PCO_2_ (mmHg; mean ± SD)	58.9 ± 9.8

*FEV1* forced expiratory volume in 1 s, *FVC* forced vital capacity, *Rtot* resistance, *RV* residual volume, *TLC* total lung capacity, *SD* standard deviation

**Fig. 2:**
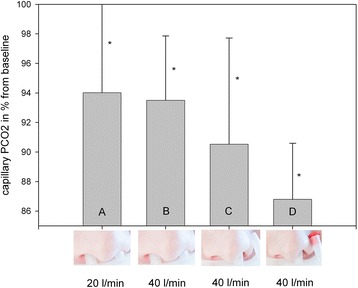
Percentage decrease in capillary PCO_2_ in COPD patients after 1 h of NHF breathing under different experimental conditions. * = *p* < 0.05 vs. baseline; baseline PCO_2_ > 55 mmHg, **a**: *n* = 14, **b**: *n* = 11, **c**: *n* = 9, **d**: n = 9

A total of 26 out of 36 patients recruited for PCO_2_ measurements had a baseline PCO_2_ > 55 mmHg. In this hypercapnic subgroup of COPD patients, PCO_2_ was significantly reduced by all conditions (A-D). The reduction of capillary PCO_2_ by condition A was more pronounced when the flow rate was increased from 20 to 40 L/min (condition A = > B). The increase of leakage and the reduction of the effective flow rate (by using only one nostril) to 20 L/min (condition C) resulted in an even greater decrease. Yet, the largest reduction of capillary PCO_2_ was obtained after increasing the flow rate in a single prong to 40 L/min by occluding the prong that was located outside the nostrils (condition D). Between conditions A and D, there was an additional significant decrease in PCO_2_ (Table 2, Fig. 2). There was no significant difference between the capillary PCO_2_ values obtained after 30 min and those obtained after 60 min of NHF therapy.

**Table 2:** Changes of mean airway pressure and PCO_2_ in patients with baseline PCO_2_ > 55 mmHg under different experimental conditions

				
Condition	A	B	C	D
Mechanically gene-rated flow rate (L/min)	20	40	40	40
Effective nasal flow rate (L/min)	20	40	20	40
Mean airway pressure (mbar ± SD)	0.57 ± 0.38 *p* = 0.03	2.3 ± 1.6 *p* = 0.02	1.63 ± 1.4 *p* = 0.08	2.13 ± 1.7 *p* = 0.05
PO_2_ (% baseline PO_2_ ± SD)	99.2 ± 18.9 *p* = 0.2	93.1 ± 15.7 *p* = 0.18	93.8 ± 17.6 *p* = 0.12	93.5 ± 11.9 *p* = 0.05
PCO_2_ (% baseline PCO_2_ ± SD)	94.2 ± 8.3 *p* = 0.01	93.5 ± 4.4 *p* = 0.000	90.5 ± 7.2 *p* = 0.005	86.8 ± 3.8 *p* = 0.001

All values are mean ± SD. *SD* standard deviation

**Fig. 3:**
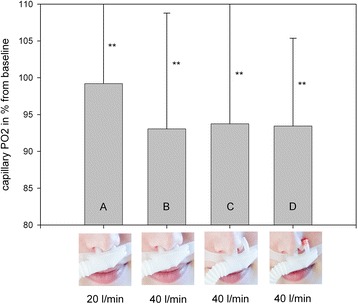
Percentage decrease of capillary PO_2_ in COPD patients after 1 h of NHF breathing under different experimental conditions. ** = *p* > 0.05 vs. baseline; baseline PCO_2_ > 55 mmHg, **a**: *n* = 14, **b**: *n* = 11, **c**: *n* = 9, **d**: *n* = 9

In all measurements, capillary PO_2_ did not differ significantly although a trend to lower values was observed under conditions B to D (Fig. 3). PH increased significantly under conditions B, C, and D.

**Fig. 4:**
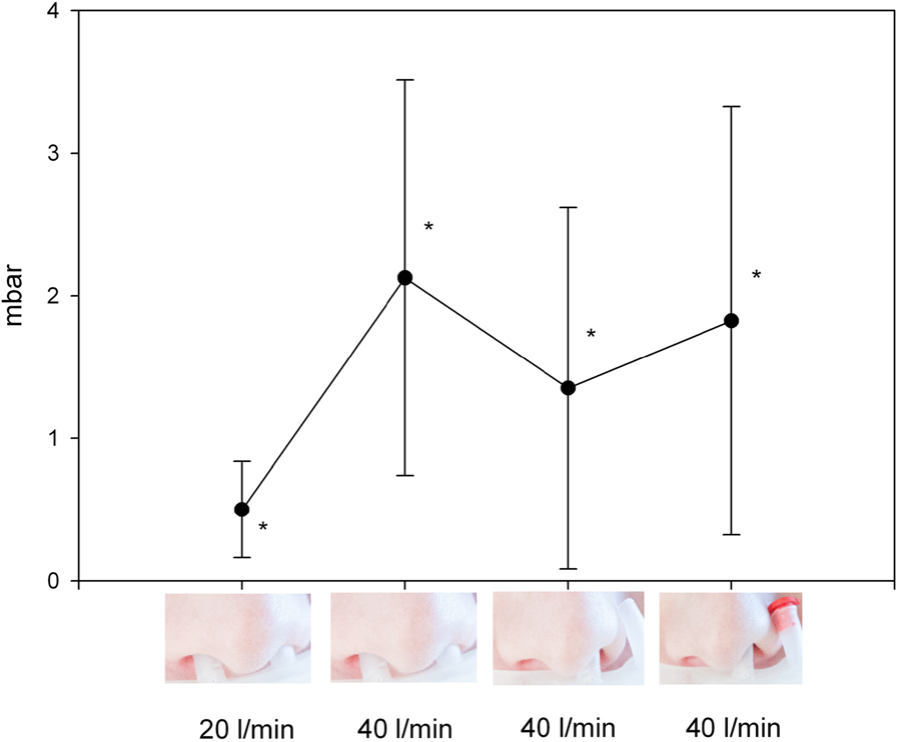
Changes in mean airway pressure in COPD patients under different experimental conditions. * = *p* < 0.05 vs. baseline; *n* = 10

Mean airway pressure was measured in 10 patients (Table 2, Fig. 4). In contrast to the above-mentioned results, mean airway pressure achieved its maximum with condition B (Fig. 4). Significant pressure differences to baseline were only observed with conditions A and B.

## Discussion

In this study, we found a leakage and flow dependent decrease of hypercapnia in COPD patients by NHF treatment. These data support the assumption that elimination of CO_2_ during NHF therapy is independent of an increase of mean airway pressure.

NHF therapy is increasingly investigated for use in clinical practice. Its benefits have been demonstrated in the treatment of patients with acute respiratory insufficiency (ARI) due to pulmonary causes and in the prevention of postextubation respiratory failure [3–7]. In addition, smaller reports have suggested the use of NHF therapy in other medical indications [8–11]. Potential NHF applications are still to be defined, and the NHF mechanism of action needs to be further elucidated. In particular, the effects on oxygenation, hypercapnia, and dyspnea need to be characterized [3, 9].

Little is known about the mechanisms that underlie NHF therapy. Previous studies have demonstrated increases in airway pressure [8] but the changes in airway pressure were small. In our study, mean airway pressure increased with NHF therapy, confirming previous results [8, 10]. Increase of flow rates resulted in an increase in airway pressure. It achieved its maximum under the conditions of setting B where gas was supplied through both nostrils (low leakage condition) with the highest flow rate tested (40 L/min). Mean airway pressure appeared to depend on the flow rate since doubling the effective flow rate (A = > B, C = > D) led to increased mean airway pressures in both cases. However, the mean airway pressure also depended on the degree of leakage (B vs. D), in line with the notion that pressure is released through leaking nostrils. Thus, in addition to the confirmation that NHF therapy leads to a flow dependent increase in mean airway pressure, our results indicate that the increase in mean airway pressure also depends on the degree of leakage, or vice versa to the extent to which the upper airways are occluded (nostrils, mouth). In adults, the nostrils are only partially occluded by the prongs, whereas in neonates, the prongs can completely obstruct the nostrils, which leads to a larger increase in mean airway pressure, rendering NHF treatment more similar to nCPAP therapy [2]. This signifies a fundamental difference between the ways in which NHF therapy works in neonates and adults.

If the increase in airway pressure was the principal reason accounting for the clinical effects of NHF therapy in adults, capillary PCO_2_ should achieve its minimum when mean airway pressure rises to its maximum, but this is not what was observed in this study. In contrast, in our study, PCO_2_ achieved its minimum with condition D, whereas mean airway pressure reached its maximum with condition B. Increased leakage (B = > C) resulted in a greater decrease in PCO_2_ that went along with a decrease of mean airway pressure, demonstrating that the reduction in PCO_2_ is not due to an increase in mean airway pressure. Rather, it relies on a higher degree of leakage and a concomitant airway wash out. This wash out affects the upper airways, as shown by Möller et al. who demonstrated CO_2_ elimination from nasal dead space and reduction of CO_2_-rebreathing under NHF therapy [16]. A model study by Bräunlich et al. [17] indicated that CO_2_ wash out may extend to the lower airways. Our data confirm previous data obtained by Frizzola et al. who demonstrated that the reduction of PCO_2_ with NHF therapy in piglets similarly depends on the degree of leakage [18].

As shown in this study, oxygen saturation tended to slightly decrease when using higher flow rates and constant oxygen supplementation. This observation is not surprising because FIO_2_ decreased by applying higher amounts of room air and stable oxygen supplementation. We aimed at integrating this observation into our study because of both insufficient literature data and the hypothesis that reduction in oxygen can lead to a reduction in PCO_2_. Interestingly, there was no change in PO_2_ under low flow/low leakage of condition A. This study supports the observed necessity to increase rates of supplemented oxygen to keep FIO_2_ constant at higher flow rates. The non-significant decrease in oxygen cannot be the reason for decreased PCO_2_ levels in this study because of (1) constant oxygen values in different settings and regardless decreasing PCO_2_ values (conditions B, C, D) [10], (2) stable PO_2_ and decreased PCO_2_ values when using 20 L/min (condition A) [8, 10, 12, 21], and (3) documented reduction of respiratory rates despite of PO_2_ reduction [8, 10, 22].

NHF therapy partially relieves the work of breathing in COPD patients [10, 13, 21]. This is also evidenced from the increase in tidal volume and concomitant decrease in respiratory rate as well as the reduction in minute volume as previously reported [10]. In hypercapnia, respiratory muscle function deteriorates, resulting in reduced tidal volumes and higher respiratory rates. This, in turn, leads to a decrease of alveolar ventilation. Dead space ventilation increases in proportion to overall ventilation. By constantly placing the air/oxygen mixture in the airways, functional dead space is reduced and tidal volume can adjust and, accordingly, declines. If kept constant, alveolar ventilation is increased. Together with the wash out of airways, NHF is able to work as a ventilation support device.

### Study limitations

Our study has three limitations. Since experiments were time consuming and some patients tolerated only few measurements, it was not possible to test all four conditions within the clinical setting. Bias cannot entirely be excluded because patients as well as investigators could not be blinded regarding the different experimental conditions. The fact that only COPD patients were tested could limit the generalizability of our findings.

## Conclusions

In the present study, we showed that NHF therapy significantly reduces capillary PCO_2_ in COPD patients. Higher flow rates and increased leakage of the upper airways appear to increase the effectiveness in reducing PCO_2_. At the same time, an increase in airway pressure is not required. Our data indicate that airway wash out is the major mechanism of action in NHF breathing.

## Abbreviations

(n) CPAP: (nasal) continuous positive airway pressure
ANOVA: Analysis of variance
ARI: Acute respiratory insufficiency
BGA: Blood gas analysis
COPD: Chronic obstructive pulmonary disease
NHF: Nasal highflow
PCO_2_: Partial pressure of CO_2_
PO_2_: Partial pressure of O_2_
